# Nebulized exosomes derived from allogenic adipose tissue mesenchymal stromal cells in patients with severe COVID-19: a pilot study

**DOI:** 10.1186/s13287-022-02900-5

**Published:** 2022-05-26

**Authors:** Ying-Gang Zhu, Meng-meng Shi, Antoine Monsel, Cheng-xiang Dai, Xuan Dong, Hong Shen, Su-ke Li, Jing Chang, Cui-li Xu, Ping Li, Jing Wang, Mei-ping Shen, Cheng-jie Ren, De-chang Chen, Jie-Ming Qu

**Affiliations:** 1 Department of Pulmonary and Critical Care Medicine, Hua-Dong Hospital, Fudan University, 221, West Yan’an Rd., Shanghai, 200040 China; 2 Department of Pulmonary and Critical Care Medicine, Rui-Jin Hospital, Shanghai Jiao-Tong University School of Medicine, 197, Rui Jin Er Rd., Shanghai, 200025 China; 3 Institute of Respiratory Disease, Shanghai Jiao-Tong University School of Medicine, Shanghai, China; 4 Department of Intensive Care Unit, Rui-Jin Hospital, Shanghai Jiao-Tong University School of Medicine, Shanghai, China; 5 Multidisciplinary Intensive Care Unit, Department of Anesthesiology and Critical Care, La Pitié-Salpêtrière Hospital, Assistance Publique-Hôpitaux de Paris (APHP), Sorbonne University, Paris, France; 6 INSERM, UMR S959, Immunology-Immunopathology- Immunotherapy (I3), Sorbonne Université, 75005 Paris, France; 7 Biotherapy (CIC-BTi) and Inflammation-Immunopathology-Biotherapy Department (DHU i2B), Hôpital Pitié-Salpêtrière, AP-HP, 75651 Paris, France; 8 Cellular Biomedicine Group Inc. (CBMG), Shanghai, China; 9 Department of Pulmonary and Critical Care Medicine, Wuhan Jinyintan Hospital, Wuhan, China; 10 Key Laboratory of Emergency Prevention, Diagnosis and Treatment of Respiratory Infectious Diseases, Shanghai, China; 11 Daxing Research Institute, University of Science and Technology Beijing, Beijing, China

**Keywords:** COVID-19, Mesenchymal stromal cell, Exosomes, Inhalation, Extracellular vesicles

## Abstract

**Background:**

Existing clinical studies supported the potential efficacy of mesenchymal stromal cells as well as derived exosomes in the treatment of COVID-19. We aimed to explore the safety and efficiency of aerosol inhalation of the exosomes derived from human adipose-derived MSCs (haMSC-Exos) in patients with COVID-19.

**Methods:**

The MEXCOVID trial is a phase 2a single-arm, open-labelled, interventional trial and patients were enrolled in Jinyintan Hospital, Wuhan, China. Eligible 7 patients were assigned to receive the daily dose of haMSCs-Exos (2.0 × 10^8^ nano vesicles) for consecutively 5 days. The primary outcomes included the incidence of prespecified inhalation-associated events and serious adverse events. We also observed the demographic data, clinical characteristics, laboratory results including lymphocyte count, levels of D-dimer and IL-6 as well as chest imaging.

**Results:**

Seven severe COVID-19 related pneumonia patients (4 males and 3 females) were enrolled and received nebulized haMSC-Exos. The median age was 57 year (interquartile range (IQR), 43 year to 70 year). The median time from onset of symptoms to hospital admission and administration of nebulized haMSC-Exos was 30 days (IQR, 15 days to 40 days) and 54 d (IQR, 34 d to 69 d), respectively. All COVID-19 patients tolerated the haMSC-Exos nebulization well, with no evidence of prespecified adverse events or clinical instability during the nebulization or during the immediate post-nebulization period. All patients presented a slight increase of serum lymphocyte counts (median as 1.61 × 10^9^/L vs. 1.78 × 10^9^/L). Different degrees of resolution of pulmonary lesions after aerosol inhalation of haMSC-Exos were observed among all patients, more obviously in 4 of 7 patients.

**Conclusions:**

Our trial shows that a consecutive 5 days inhalation dose of clinical grade haMSC-Exos up to a total amount of 2.0 × 10^9^ nano vesicles was feasible and well tolerated in seven COVID-19 patients, with no evidence of prespecified adverse events, immediate clinical instability, or dose-relevant toxicity at any of the doses tested. This safety profile is seemingly followed by CT imaging improvement within 7 days. Further trials will have to confirm the long-term safety or efficacy in larger population.

*Trial Registration*: MEXCOVID, NCT04276987.

## Background

Coronavirus disease 2019 (COVID-19), caused by severe acute respiratory syndrome coronavirus 2 (SARS-CoV-2), has been rampant across the globe for more than two years and so far killing over five million people. Mesenchymal stromal cells (MSCs) and their derived extracellular vesicles (EVs) are a potential treatment for COVID-19 due to their capability to modulate the immune response, promote pathogen clearance and mitigate the severity of organ injuries.

Many clinical studies have demonstrated that mesenchymal stromal cells (MSCs) and their derived exosomes (MSCs-Exo) significantly reduced lung inflammation resulting from different types of lung injury. A study of 11 patients with COVID-19-associated ARDS showed that intravenous infusion (a total of 60 × 10^7^ cells) of human umbilical cord MSCs (UC-MSCs) or placental MSCs (PL-MSCs) rapidly improved respiratory distress along with reducing the excessive inflammatory response [1]. Several randomized, double-blind, placebo-controlled trials suggested that UC-MSC (a total of 10–12 × 10^7^ cells) promoted recovery of lung lesion in COVID-19 patients without safety risk [2–5]. However, the small sample-size of patients included in the trials, the important heterogeneity in the severity of the included patients, the tissue source, the therapeutic doses, the timing of the cells administration, the percentage of viability, the bioactivity and the inter-batch variability of the MSCs, are all methodological limitations that preclude drawing any definitive conclusions about the efficacy of MSCs in this indication. Furthermore, intravenous MSCs-based therapy-related issues include the potential risk of mutagenicity and oncogenicity, the uncertainty about the wide range of MSCs viability after preparation for infusion and the optimized methods for cryopreservation, thawing, and production of MSCs [6, 7].

MSCs-Exo, on the other hand, own similar therapeutic properties to MSCs in lung injury models, with more accessibility to be prepared, stored, and delivered to the bedside while circumventing certain limitations and caveats inherent to using parent cells. So far, most published clinical trials about MSCs as well as MSCs-Exo focusing on COVID-19 associated ARDS were administered intravenously. In these studies, although the safety profile of MSCs and MSC-EVs treatment was suggested to be correct, the nonsignificant therapeutic effect might lie in the route of administration. The nebulized route constitutes a particularly interesting route of administration in the context of lung damage, given its excellent performance in terms of the bioavailability of the drug delivered to the targeted pulmonary site [8]. To date, studies evaluating the efficacy of clinical-grade MSCs-derived exosomes remain sparse, and the feasibility of nebulized route of administration has never been investigated, even though attractive in the context of severe SARS-CoV-2-induced pneumonia.

Given the severe situation of COVID-19 worldwide, we aimed to assess the safety of aerosol inhalation of the exosomes derived from human adipose-derived MSCs (haMSCs-Exo) in the treatment of patients with severe COVID-19 related pneumonia, to explore the optimum dosage as well as delivery route of MSCs-based therapy for acute respiratory diseases.

## Methods

### Study design and participants

The phase 2a single-arm, open labelled, interventional clinical trial MEXCOVID study (NCT04276987) was approved by the Ethics Commission of Jinyintan hospital as well as Rui-jin Hospital, Shanghai Jiao-tong University School of Medicine, Shanghai, China, and conducted at Jinyintan hospital, Wuhan, China, starting enrollment from March 16th, 2020. The inclusion criteria included 1) ages are differing from 18 years old to 75 years old, 2) confirmed infection with SARS-CoV-2 with PCR, 3) according to the fifth version of the guidelines on the Diagnosis and Treatment of COVID-19 by the National Health Commission, COVID-19 severity was classified as severe or critical type. The full inclusion and exclusion criteria are shown in Table 1. Due to the restricted accessibility during the epidemic period, all the candidates had already been admitted to the ICU and received antiviral therapy and other supportive care, while some patients received antibiotic treatment, antifungal treatment, glucocorticoid, and oxygen support at the appropriate situation. All the eligible patients met the criteria by the day of enrollment, one day before haMSC-Exos administration. Written Informed consent was obtained after discussion with patient or an appropriate surrogate. The process was carried out within the isolation units at Jinyintan hospital. Senior doctors (XD, HS and DCC) were responsible for introducing the protocol to the candidates and all the participants were voluntary to sign the informed consent with the presence of DCC and HS with their signatures simultaneously. All the documents were recorded by taking photographs. A total of seven patients received the initial dose of haMSC-Exos (2.0 × 10^8^ particles per day) for five consecutive days (total cumulative therapeutic dose of 1.0 × 10^9^ haMSC-Exos per patient), based on the well tolerated dose of haMSCs-Exo from MEXVT study (NCT04313647). Data from the first patient were reviewed for safety before proceeding with an enrollment of next patients.

**Table 1:** The eligibility criteria of the MEXCOVID-19 study

*Inclusion criteria*
1. The subjects or their family members voluntarily participated in the study and signed the informed consent 2. Ages are differing from 18 years old to 75 years old 3. Confirmed infection with SARS-CoV-2 4. According to the fifth version of the guidelines on the Diagnosis and Treatment of COVID-19 by the National Health Commission, COVID-19 severity is classified as severe or critical type: *Severe type:* (1) Respiratory, distress, respiratory rate 30 per minute (2) Oxygen saturation on ambient air at rest ≤ 93% (3) Partial pressure of oxygen in arterial blood/ fraction of inspired oxygen ≤ 300 mmHg *Critical type:* (1) Respiratory failure occurs, and mechanical ventilation is required (2) Shock occurs (3) Patients with other organ dysfunction needing intensive care unit monitoring treatment
*Exclusion criteria*
1. Patients with severe allergy history 2. Pneumonia caused by bacteria, mycoplasma, chlamydia, Legionella, fungi, parasites, or other viruses 3. HAP/VAP (hospital-acquired pneumonia/ventilator-acquired pneumonia) caused by lung cancer or other known reasons 4. Suffering from carcinoid tumor or carcinoid syndrome 5. Recent use of immunosuppressive drugs 6. History of epilepsy, needing continuous anticonvulsant therapy, or having received anticonvulsant therapy in the past three years 7. History of severe chronic lung diseases or requiring long-term home oxygen therapy 8. Undergoing hemodialysis or peritoneal dialysis 9. According to the local laboratory values, the creatinine clearance rate less than 15 ml/min 10. Moderate or severe hepatic failure (child Pugh score > 12) 11. Expecting to receive any of the following drugs during the study period: valproic acid or sodium dipropionate used within 2 weeks before screening; 5-tryptamine reuptake inhibitors, tricyclic antidepressants, 5-HT1 receptor agonists (triptans), or monoamine oxidase inhibitors (or MAOIs used within 2 weeks before screening) 12. Cannot understand and implement the investigation plan 13. Suffering from lower extremities deep venous thrombosis or pulmonary embolism in the past 3 months 14. Undergoing ECMO or high-frequency oscillatory ventilation 15. People with HIV, hepatitis virus, or syphilis 16. Pregnant or nursing females 17. According to the judgment of the researcher, the one who has a low probability of being included in the group (such as frailty, etc.)

Clinical-grade human adipose-derived MSCs-Exosomes (haMSC-Exos) were prepared from Cellular Biomedicine Group, Inc. (CBMG, Shanghai, China, https://www.cellbiomedgroup.com) and the detailed process of manufacture and quality control of haMSC-Exos were presented in our previous articles [9]. haMSC-Exos were prepared from CBMG and shipped frozen, from Shanghai to Wuhan, directly to the clinical site in a validated dry ice shipper with a continuous temperature monitoring device. Upon receipt, the Exos solution was inspected and stored in a controlled, continuously monitored in − 20 °C storage tank within the isolation units. All the handovers as well as signature documents were recorded by taking photographs. Prior to administration, the solutions were thawed, reconstituted at the clinical site.

### Procedures

The Data Safety Monitoring Group (DSMG) was comprised of critical care physicians with MEXVT trial experience and was responsible for reviewing data for each patient and making recommendations regarding continuing, stopping, or altering the trial. The skin test of haMSC-Exos was performed before the first inhalation as described [9]. Starting from the morning of Day 1, inhalation of haMSC-Exos was administered using a mesh nebulizer set (Aerogen Solo system, Ireland) at 9am each day with a total volume of 6 ml diluted with normal saline for 30 min for five consecutive days. All patients were monitored closely for any changes in a prescribed list of temperature, respiratory or cardiovascular parameters. Follow-up laboratory tests such as white blood cell count, lymphocyte count, chemistry panels assessing liver and kidney function, C-reactive protein (CRP), lactate dehydrogenase (LDH), interleukin 6 (IL-6), and CT scan were collected at baseline and after the cumulative dose of inhalation treatment. The incidence and nature of all serious adverse events were reviewed and independently evaluated by the DSMG to determine whether they were thought to be related to haMSC-Exos inhalation, with a particular focus on events that would be unexpected in COVID-19.

### Clinical outcomes

The primary objectives were to assess the safety and tolerability. We recorded the vital signs of all the participants at the different periods before and after inhalation. Meanwhile, we reported the incidence of all serious adverse events, including death, and the incidence of prespecified inhalation-associated events, such as fever, shortness of breath, diarrhea and epilepsy, etc., and non-serious adverse events thought to be related to the nebulization process. Clinical information for the patients before and after 5-day inhalation treatment was obtained from a review of the hospital computer medical system and included the following: 1) demographic data, principal symptoms, days of admission from symptom onset, and comorbidity; 2) various therapeutic data, including mechanical ventilation, antiviral therapies, antiviral or antifungal therapies, steroids, and convalescent plasma (CP) therapy. CP transfusion defined as one dose of 200 mL of inactivated CP derived from recently recovered donors with the neutralizing antibody titers above 1:640 was transfused to the patients within 4 h as an addition to maximal supportive care and antiviral agents [10]. All the candidates were enrolled in CP transfusion unless they had previous allergic history to plasma or ingredients (sodium citrate), or severe organ dysfunction, who were not suitable for CP transfusion; 3) laboratory data, including white blood cell count, lymphocyte count, chemistry panels assessing liver and kidney function, CRP, LDH, IL-6 and; 4) chest imaging scoring data.

Regarding the CT score, all CT images were reviewed by two independent radiologists using a viewing console. Decisions were reached by consensus. Each segment of the lung was reviewed for opacification, and the lesion size was described as small (diameter < 1 cm), medium (diameter, 1 to < 3 cm), large (diameter, 3 cm to < 50% of the segment), or segmental (> 50% of the segment), scored as 1 to 4 point, respectively [11]. The lesion was assessed segment by segment, and the total score ranged up to 72 points. The form of the lesion (mainly ground-glass opacity and consolidation) was classified as patchy or oval according to its shape on serial images.

### Statistical analysis

Continuous variables were presented as median and 25–75th interquartile range (IQR). CT score before and after inhalation treatment were compared using Wilcoxon test (nonparametric equivalent of the paired t test). Systemic clinical outcomes and biomarker values were compared using Kruskal–Wallis tests. All statistical analysis was performed using GraphPad Prism software (La Jolla, California, USA). Remaining analyses are descriptive.

## Results

### Clinical characteristics of participants

From March 16th, 2020 to March 25th, 2020, seven severe COVID-19 patients (4 males and 3 females) were enrolled and received aerosol inhalation of haMSC-Exos. The median age was 57 y (IQR, 43 y to 70 y) (Table 2). The median time from onset of symptoms to hospital admission and aerosol inhalation of haMSC-Exos was 30 days (IQR, 15 days to 40 days) and 54 days (IQR, 34 days to 69 days), respectively. All these seven patients had a fever at disease onset. The second common symptoms on the onset day of haMSC-Exos administration were shortness of breath (5 of 7) and cough (5 of 7). Malaise (3 patients), expectoration (1 patient), sore throat (1 patient), headache (1 patient), and diarrhea (1 patient) were less common. Five patients had underlying chronic diseases, including hypertension, diabetes, chronic obstructive pulmonary disease (COPD), and hyperthyroidism. All of them were given antiviral and antibiotic or antifungal treatment (Table 3). Besides, three patients received corticosteroid therapy, while four patients received convalescent plasma transfusion (Table 3). As of April 5th, 2020, all seven patients were discharged from the hospital.

**Table 2:** Demographic information and baseline characteristics of patients in MEXCOVID study

	Age (years)	Sex	Occupation	Allergic history	Smoking history (pack years)	Clinical classification	Days of admission from symptom onset (days)	Days of haMSCs-Exos nebulization from symptom onset (days)	Principal symptoms	Comorbidity
Patient 1	34	Male	Unemployed	Denied	400	Severe	15	32	Fever, shortness of breath	Hypertension
Patient 2	51	Female	Self-employed	Denied	None	Severe	15	59	Fever, sore throat, shortness of breath, cough, expectoration, malaise	None
Patient 3	43	Male	Employee	Denied	None	Severe	30	34	Fever, cough	Diabetes, Hypertension
Patient 4	60	Male	Employee	Denied	800	Severe	40	85	Fever, cough, malaise, shortness of breath	Chronic obstructive pulmonary disease, hypertension
Patient 5	57	Female	Unemployed	Denied	None	Severe	11	69	Fever, headache, diarrhea	Hyperthyroidism
Patient 6	75	Male	Unemployed	Denied	None	Severe	44	54	Fever, cough, shortness of breath, malaise	None
Patient 7	70	Female	Unemployed	Penicillin	None	Severe	38	47	Fever, cough, shortness of breath	Diabetes

**Table 3:** Other treatments of patients in MEXCOVID study

	Treatment received				Oxygen support	
	Antiviral treatment	Antibiotic or antifungal treatment	Corticosteroids treatment	Convalescent plasma transfusion	Before haMSCs-Exo nebulization	After haMSCs-Exo nebulization
Patient 1	Arbidol, ribavirin, IFN-α	Cefoperagone sodium and tazobactam sodium	None	Yes	Nasal cannula	Nasal cannula
Patient 2	Arbidol, ribavirin, IFN-α, lopinavir-ritonavir	Cefoperagone sodium and tazobactam sodium, meropenem	Methylprednisolone	Yes	Nasal cannula	Nasal cannula
Patient 3	Arbidol, IFN-alpha	None	None	None	Nasal cannula	Nasal cannula
Patient 4	Arbidol, oseltamivir	Cefoperagone sodium and tazobactam sodium, meropenem, moxifloxacin	Methylprednisolone	Yes	High-flow nasal cannula	Nasal cannula
Patient 5	IFN-α	Cefoperagone sodium and tazobactam sodium, meropenem	Methylprednisolone	Yes	Nasal cannula	Nasal cannula
Patient 6	Arbidol, IFN-α	Cefoperagone sodium and tazobactam sodium,	None	None	High-flow nasal cannula	Nasal cannula
Patient 7	Ganciclovir	Moxifloxacin, meropenem, fluconazole	None	None	Nasal cannula	Nasal cannula

### Clinical manifestations, laboratory and radiological findings

**Fig. 1:**
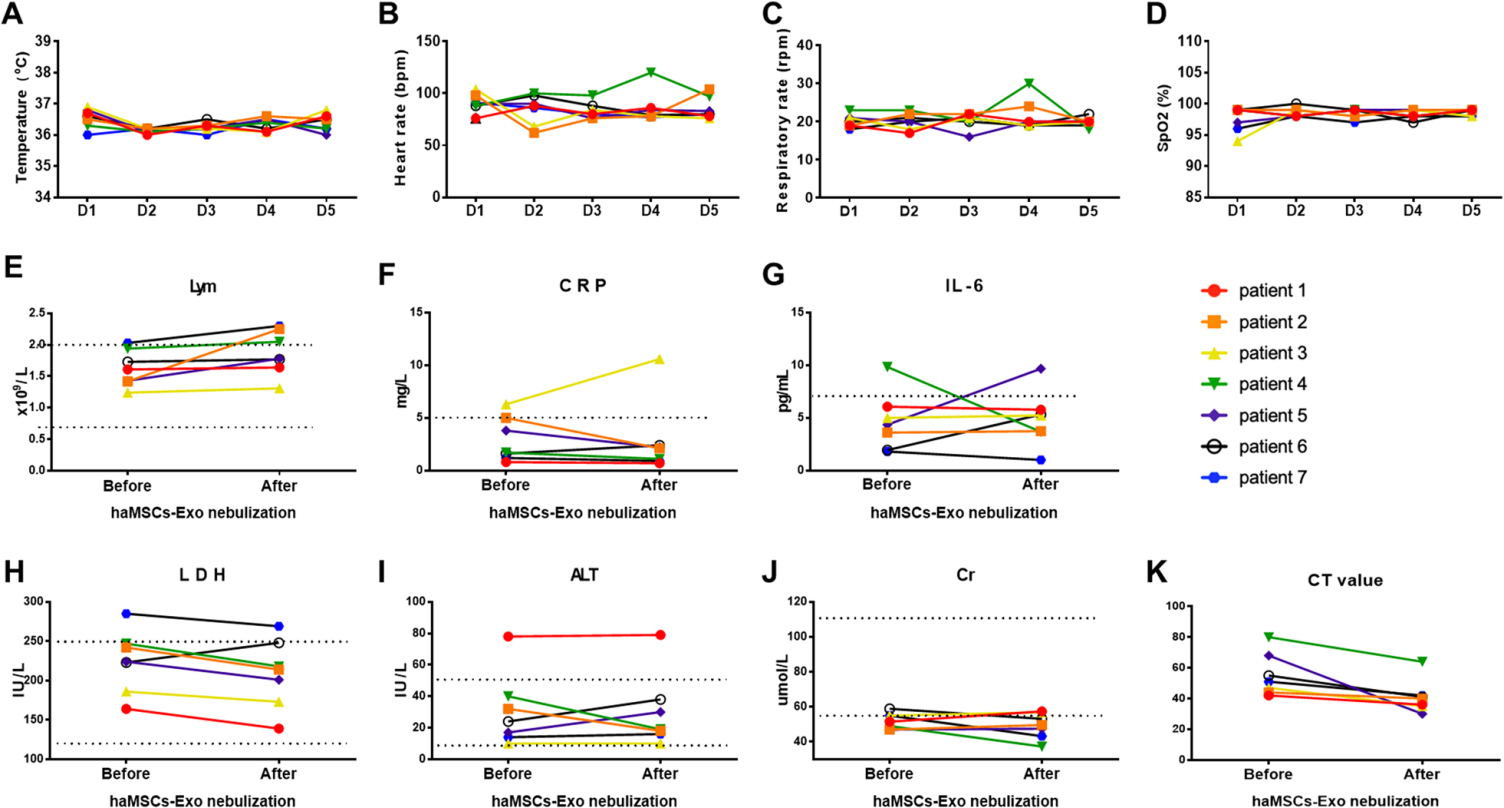
Clinical characters of patients in the MEXCOVID study. **A** Temperature **B** heart rate **C** respiratory rate **D** oxyhemoglobin saturation before and after hMSC-Exos nebulization in COVID-19 patients. **E**–**J** Dynamic changes of laboratory parameters before and after haMSC-Exos nebulization in COVID-19 patients. **K** Chest CT score before and after haMSC-Exos nebulization in COVID-19 patients. The dotted horizontal line represents the reference value range. *haMSC-Exos* human adipose-derived MSCs-Exosomes; *COVID-19* coronavirus disease 2019; *Lym* lymphocyte; *CRP* C reactive protein; *IL-6* Interleukin-6; *LDH* Lactate dehydrogenase; *ALT* alanine aminotransferase; Cr: creatinine

All of 7 COVID-19 patients tolerated the haMSC-Exos nebulization well, with no evidence of prespecified adverse events or clinical instability, aggravation of existing symptoms, during the nebulization or in the immediate post-nebulization period. The vital signs (in temperature, heart rate, respiratory rate, and saturation oxygen) of the seven patients stayed stable during the five-day aerosol inhalation course (Fig. 1A–D).

As a critical prognostic indicator of COVID-19, lymphocytopenia has been on an improving trend after aerosol inhalation of haMSC-Exos (median as 1.61 × 10^9^/L vs. 1.78 × 10^9^/L) in MEXCOVID, all seven patients showing an increase of lymphocyte counts (Fig. 1E). In terms of inflammation biomarkers, a trend towards a decrease was observed, including C-reactive protein (CRP) (a decrease found in 6 out of 7) (Fig. 1F), interleukin-6 (IL-6) (a decrease found in 5 out of 7) (Fig. 1G), lactate dehydrogenase (LDH) (a decrease found in 6 out of 7) (Fig. 1H). The alanine aminotransferase (ALT) (median as 78 IU/L vs. 79 IU/L) remained within normal range before and after aerosol treatment in patients except for patient 1 (Fig. 1I). The stable ALT and creatinine (Cr) (median as 51 μmol/L vs. 40 μmol/L) level indicated that aerosol inhalation of haMSC-Exos had no hepatotoxicity or nephrotoxicity (Fig. 1I–J). The CT score value (median as 51 points before treatment vs. 40 points after treatment,* p* = 0.0559) of these seven patients dropped after aerosol therapy (Fig. 1K).

**Fig. 2:**
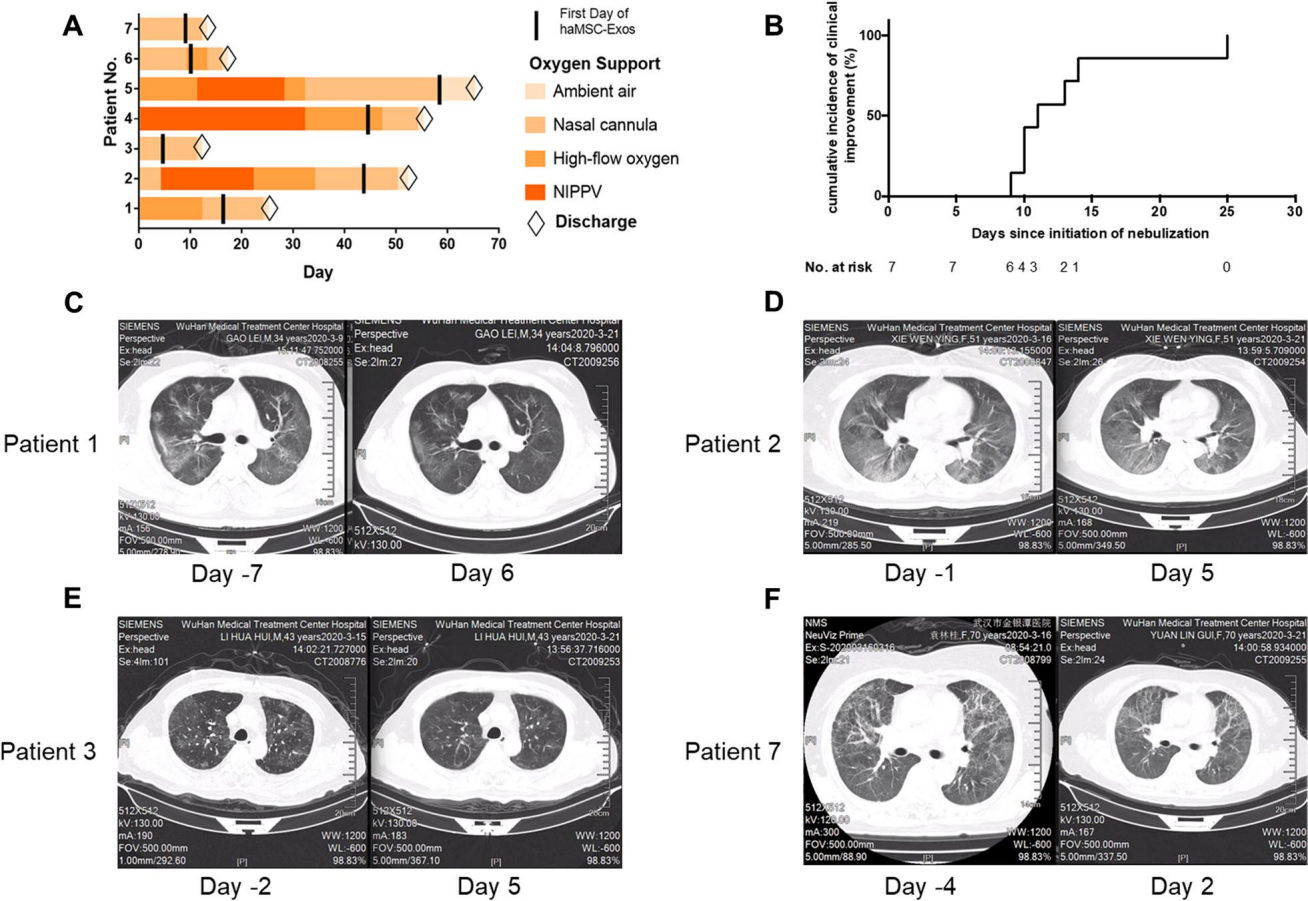
Clinical improvement from baseline, the onset day of inhalation treatment, in individual patients in the MEXCOVID study. **A** Changes in oxygen-support status from baseline in individual patients.** B** Cumulative incidence of clinical improvement from the onset of neublization.** C**–**F** Changes of chest CT scan before and after* haMSC-Exos* inhalation in COVID-19 patients

**Fig. 3:**
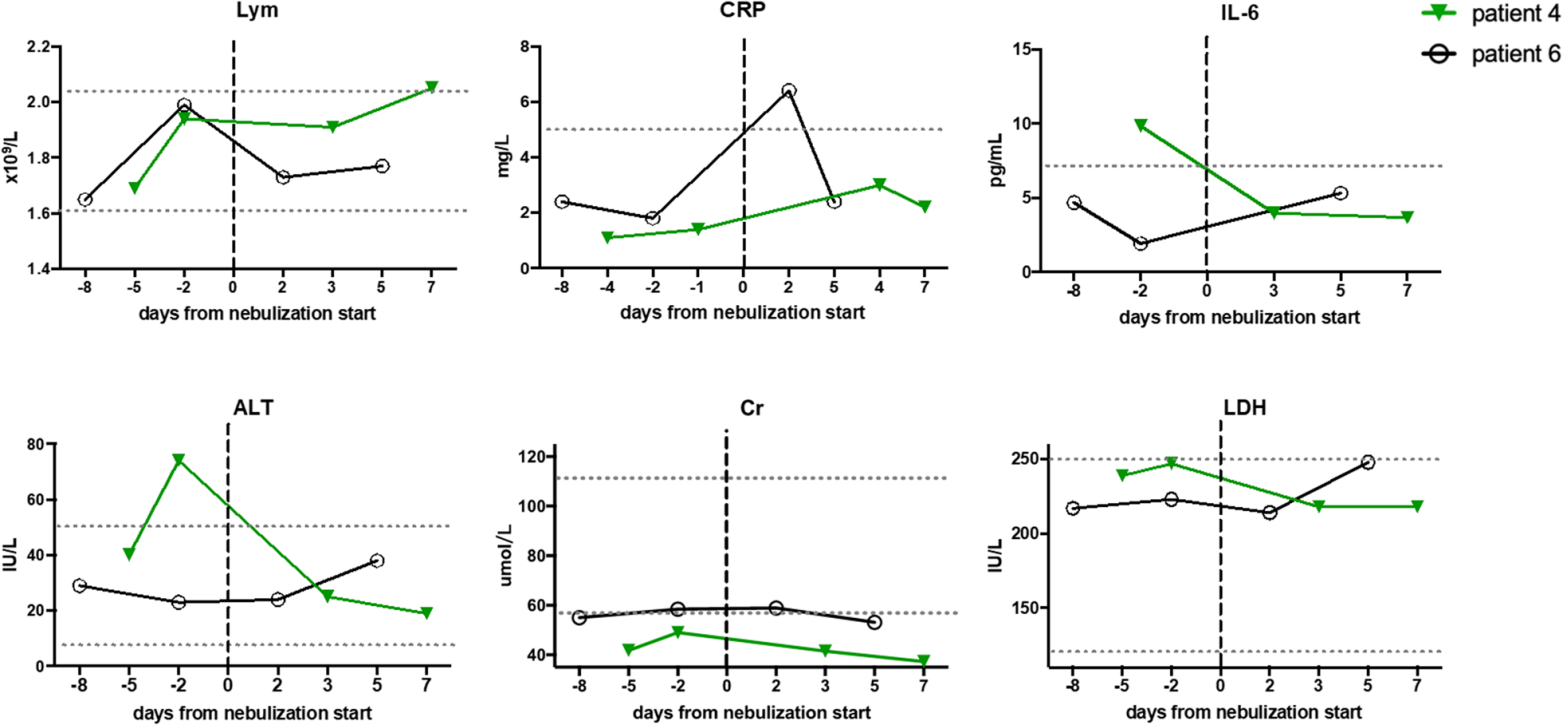
Dynamic changes of laboratory parameters in patient 4 and patient 6 before and after haMSC-Exos nebulization. The dotted horizontal line represents the reference value range. *Lym* lymphocyte; *CRP* C reactive protein; *IL-6* interleukin-6; *ALT* alanine aminotransferase; *Cr* creatinine; *LDH* lactate dehydrogenase

Of all the seven patients, patients 4 and 6 were receiving high-flow oxygen at the beginning of aerosol inhalation of haMSC-Exos, and switched to nasal cannula at the 3rd and 4th day of nebulization (Fig. 2A). Despite the change of oxygen support method in patient 4 and patient 6 before and after aerosol therapy, no striking amelioration in laboratory parameters was observed (Fig. 3).

Different degrees of resolution of pulmonary lesions after aerosol inhalation of haMSC-Exos were observed in all patients. Representative chest CT images before and after aerosol inhalation of haMSC-Exos of patient 1, patient 2, patient 3, and patient 7 are shown in Fig. 2B–E. Patient 7, a 70-year-old female hospitalized 38 days from symptom onset (dso) who received aerosol therapy since 47 dso, showed the most obvious pulmonary image improvement (Fig. 2E). Compared with the result at 43 dso, massive infiltration and ground-glass opacity disappeared on the CT image performed at 48 dso. The feedbacks from all the accessible follow-up visits are shown in Table 4.

**Table 4:** Follow-up feedbacks of patients after nebulization treatment

	Days from the onset of nebulization treatment	Clinical Symptoms	COVID-19 antibody test	Lymphocyte count	Alanine aminotransferase (ALT) (U/L)	Creatinine (Cr) (μmol/L)	Chest CT images
Patient 1	Day 20	Denied	IgM ( −), IgG ( −)	2.29 × 10^9^/L	N/A	N/A	Obvious absorption of infiltration at both lobes compared with the day of symptom onset
	Day 36	Denied	N/A	N/A	N/A	N/A	Slight absorption compared with Day 20
Patient 2	Day 41	Denied	IgM ( −), IgG ( +)	2.14 × 10^9^/L	45	53	Partial absorption of infiltration compared with the day of symptom onset
Patient 3	Day 22	Denied	N/A	N/A	N/A	N/A	Normal
	Day 59	Denied	N/A	3.72 × 10^9^/L	9	63	Normal
Patient 4	Day 17	Mild cough	N/A	N/A	N/A	N/A	Infiltration at both lobes, similar to the day of symptom onset
	Day 43	Denied	N/A	N/A	N/A	N/A	Infiltration at both lobes, similar to Day 17
Patient 5	Day 36	Denied	IgM ( −), IgG ( +)	2.52 × 10^9^/L	20	55	Slight absorption compared with the day of symptom onset
Patient 6	Day 24	Mild cough	N/A	N/A	N/A	49	N/A
Patient 7	Day 28	Denied	IgM ( −), IgG ( +)	1.82 × 10^9^/L	10	50	Obvious absorption of infiltration at both lobes compared with Day 2
	Day 52	Denied	N/A	1.97 × 10^9^/L	N/A	N/A	Slight absorption compared with Day 28

## Discussion

Given the emerging crisis of COVID-19 pandemic, it would be of great value to explore a new initiative inhalation route of haMSC-Exos-based therapy on this viral respiratory infection mainly involving terminal bronchioles in its most severe form: the ARDS. We used vibrating mesh nebulizers in all our trials because there is a body of evidence supporting the use of mesh rather than jet nebulizers [12–14]. Mass median aerodynamic diameters were slightly smaller with mesh nebulizers compared to jet nebulizers. Thus, the particle size of haMSC-Exos around 100 nm meets the nebulization requirement to reach the distal lung theoretically. Our preliminary preclinical data have determined the tolerance and efficacy by implementing a mesh nebulization system in pneumonia rodent model, showing the relative improvement in survival rate [9].

Previous findings show that inhalation administration of haMSC-Exos was well tolerated in healthy volunteers in MEXVT trial [9], with no evidence of prespecified adverse events, immediate clinical instability, or dose-limiting toxicity at any of the doses tested. The human immune system and its response to external stimuli were more complex, the mass dosage equivalence did not necessarily apply when transferring from rodents to humans. For maximum safety, we started the testing dose from a tenfold reduction (1 × 10^9^ particles per patient). Also, in our previous fluorescence uptake experimental set [9], the strongest fluorescence intensity was found at 24 h post-nebulization and then gradually decreased afterwards. We therefore determined the treatment interval starting from once per day at the fixed time of each day. The primary outcomes in this study suggested that haMSC-Exos inhalation was safe among severe COVID-19 patients. Based on continuous reviews by DSMG, none of the severe adverse events reported in COVID-19 patients in MEXCOVID trial were related to multiple administrations of haMSCs-Exos inhalation.

In the present study, most of investigated patients (5 of 7) were achieved by an improvement of clinical symptoms as well as CT image scores. Although no significant differences in biomarkers and respiratory and cardiovascular parameters were found, it remained possible that differences in baseline severity of illness confounded the secondary outcomes we recorded. For example, 2 of 7 patients have improved their respiratory status by switching from high flow nasal cannula (HFNC) to standard oxygen canula after nebulization. No statistical differences were seen in laboratory results except for a favorable shift of lymphocytes and IL-6 levels. Notably, the favorable changes observed in CT imaging even within 7 days. One possible reason would be the benefit of haMSC-Exos nebulization for COVID-19 patients, especially in those with lung infiltrates. Due to the delay of enrollment, we would not be able to exclude the potential bias that it might be the normal and spontaneous course of the disease. Although conclusions about efficacy and biomarker response are unwarranted, the consistency in the results in terms of tolerability and short-term safety is still encouraging for future clinical application.

Our trial has some limitations. First, with only seven patients, we can neither generalize our phase 2 experience, nor draw conclusions about the efficacy of haMSCs-Exo for COVID-19. Since the vibrating mesh nebulized route constituted a particularly interesting route of administration in the context of lung injury, it remained to be of great value to identify how the inhalation of Exos diffused into the airway tree with the advanced real-time tracing technologies. Second, because of several procedures such as safety and tolerance test in healthy volunteers, ethics approval and quality control of exosomes product, we have to enroll our first patient in mid-March 2020 when the epidemic of COVID-19 in Wuhan has been under control. Most of surviving severe patients have been in recovery phase. Whether a different timing of administration would have been associated with different outcomes cannot be determined. Third, all patients were treated with multiple other agents (including antiviral medications), and it is not possible to determine whether the improvement observed could have been related to therapies other than haMSC-Exos inhalation. Last, since daily SARS-CoV-2 nucleic acid tests were not available in all patients, the dynamics of the viremia of SARS-CoV-2 remained unclear. The optimal timing for nebulized administration of haMSCs-Exo, therefore, needs to be determined in the future.

## Conclusions

Our trial shows that a consecutive 5 days inhalation dose of clinical grade haMSC-Exos up to a total amount of 2.0 × 10^9^ nano vesicles was feasible and well tolerated in seven COVID-19 patients, with no evidence of prespecified adverse events, immediate clinical instability, or dose-relevant toxicity at any of the doses tested. This safety profile is seemingly followed by CT imaging improvement within 7 days. Further trials will have to confirm the long-term safety or efficacy in larger population.

## Data Availability

The datasets used and/or analysed during the current study are available from the corresponding author on reasonable request.

## Abbreviations

haMSC-Exos: The exosomes derived from human adipose-derived MSCs
IQR: Interquartile range
COVID-19: Coronavirus disease 2019
SARS-CoV-2: Severe acute respiratory syndrome coronavirus 2
MSCs: Mesenchymal stromal cells
EVs: Extracellular vesicles
MSCs-Exo: Mesenchymal stromal cells derived exosomes
UC-MSCs: Human umbilical cord MSCs
PL-MSCs: Placental MSCs
DSMG: Data Safety Monitoring Group
CRP: C-reactive protein
LDH: Lactate dehydrogenase
IL-6: Interleukin 6
CP therapy: Convalescent plasma therapy
COPD: Chronic obstructive pulmonary disease
ALT: Alanine aminotransferase
Cr: Creatinine
dso: Days from symptom onset
HFNC: High flow nasal cannula
